# Sanguinarine disrupts the colocalization and interaction of HIF‐1α with tyrosine and serine phosphorylated‐STAT3 in breast cancer

**DOI:** 10.1111/jcmm.15056

**Published:** 2020-02-17

**Authors:** Qi Su, Jingjing Wang, Mengying Fan, Mohsin Ahmad Ghauri, Asmat Ullah, Bo Wang, Bingling Dai, Yingzhuan Zhan, Dongdong Zhang, Yanmin Zhang

**Affiliations:** ^1^ School of Pharmacy Health Science Center Xi’an Jiaotong University Xi’an 710061 P.R. China

**Keywords:** Breast cancer, Hypoxia‐inducible factor‐1α, Sanguinarine, Signal transducer and activator of transcription‐3

## Abstract

Breast cancer is one leading cause of death in females, especially triple‐negative breast cancer (TNBC). Hypoxia is a key feature leading to tumour progression driven by hypoxia‐inducible factor (HIF)‐1α. The aim is to investigate the mechanism of HIF‐1α and signal transducer and activator of transcription‐3 (STAT3) interaction and discover a compound to disrupt the interaction in breast cancer cells. The regulation pattern of HIF‐1α and STAT3 was analysed in hypoxic TNBC cells and patient samples. The effects of a natural alkaloid, sanguinarine, on HIF‐1α and STAT3 colocalization and interaction were evaluated in vitro and mouse xenograft models. We observed strong colocalization of HIF‐1α, p‐STAT3‐Tyr and p‐STAT3‐Ser in TNBC patient samples. Sanguinarine could inhibit the nuclear colocalization and interaction of HIF‐1α with p‐STAT3‐Tyr and p‐STAT3‐Ser in vivo and in vitro. Our results may bring insights to the HIF‐1α/STAT3 interaction in breast cancers and suggest sanguinarine as a promising candidate for HIF‐α/STAT3 inhibition.

## 1 INTRODUCTION

Breast cancer is the most diagnosed and the leading cause of cancer‐related death in women globally.^1^ Triple‐negative breast cancer (TNBC) characterized by absence of the ER, PR and HER2 has the worst prognosis.^2^ Substantial studies have identified potentially actionable molecular targets, but currently available treatments for TNBC are still limited to chemotherapy, rather than targeted therapies.^3^

Hypoxia indicates a poor clinical outcome in breast cancer.^4^ Reduced oxygen activity elevates the activity of hypoxia‐inducible factor‐1α (HIF‐1α) which is degraded under normoxia. As a transcription factor, stabilized HIF‐1α forms heterodimers and translocates to the nucleus, thereby binding to HIF responsive elements and activating target genes,^5^ indicating that targeting upstream HIF‐1α and its cofactors could be a potential treatment option for TNBC.

Constitutively activated STAT3 is another feature of solid malignancies.^6^ STAT3 pathway orchestrates signals transmitted from a certain number of cytokines and growth factors. Activation of Janus kinases leads to the tyrosine phosphorylation of STAT3, which is followed by translocation and initiation of gene transcription in the nucleus. Beside tyrosine residues, STAT3 could be serine phosphorylated, which is less well defined.^7^ Recent studies revealed that hypoxia leads to the activation of phospho‐STAT3‐Tyr, and STAT3 has been suggested to cooperate with HIF‐1α in VEGF activation under hypoxia in cancer cells.^8^ However, the colocalization and interaction of HIF‐1α/p‐STAT3‐Tyr/p‐STAT3‐Ser have yet to be investigated in breast cancer. Additionally, the discovery of small molecular compounds interfering with the cooperation of HIF‐1α/STAT3 may gain more insights to the clinical treatment for breast cancer.

Sanguinarine, a benzophenanthridine alkaloid, exhibits broad‐spectrum anticancer activities,^9^ but little attention has been paid to its effects on hypoxia‐induced breast cancer progression in breast cancers.

In this study, we found that in line with expression pattern in TNBC patient samples, hypoxia increased HIF‐1α levels and STAT3 phosphorylation at tyrosine and serine residues in TNBC cells. Sanguinarine could effectively disrupt HIF‐1α/STAT3 colocalization and interaction.

## 2 MATERIALS AND METHODS

Human TNBC MDA‐MB‐231 cells were treated with CoCl_2_ or incubated in 1% O_2_ conditions with sanguinarine. Protein localization and expression were analysed by Western blotting, immunofluorescence, co‐immunoprecipitation, etc. MDA‐MB‐231 mice xenografts were administered with sanguinarine, and protein localization was analysed by immunofluorescence. The study has been approved by the biomedical ethical committee of Health Science Center, Xi'an Jiaotong University. Detailed Material and Methods can be found in the Supplementary file.

## 3 RESULTS

### 3.1 Hypoxia promotes STAT3 activation and colocalization in breast cancer cells

**Figure 1:**
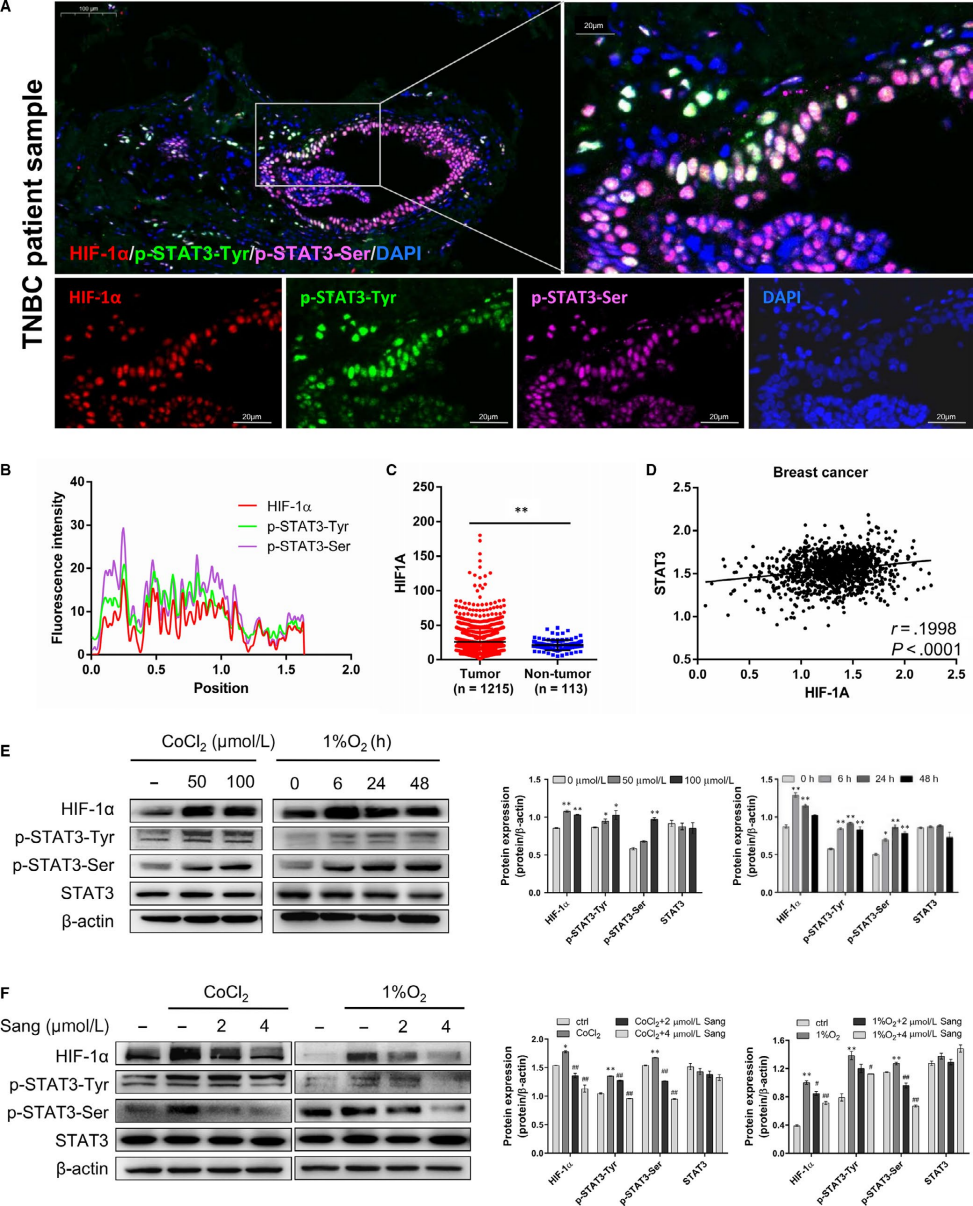
HIF‐1α/STAT3 activation and colocalization in breast cancer cells. A, HIF‐1α (red), p‐STAT3‐Tyr (green), p‐STAT3‐Ser (magenta), DAPI (blue) staining and merged and single images indicated the nuclear colocalization. B, Plot profile of Figure 1A analysed by ImageJ. C, HIF1A expression in breast cancer and normal breast tissues based on TCGA dataset (**P < .01). D, The positive correlation between the expression of HIF1A and STAT3. Data are represented as mean ± SEM. E, MDA‐MB‐231 cells were treated with indicated concentrations of CoCl_2_ for 24 h or incubated in 1% O_2_ for indicated time. HIF‐1α, p‐STAT3‐Tyr, p‐STAT3‐Ser and STAT3 protein levels were determined by Western blotting. Quantification plots are shown on the right. F, MDA‐MB‐231 cells were treated with different concentrations of sanguinarine in the absence or presence of CoCl_2_ or in the absence or presence of 1% O_2_ for 24 hours. HIF‐1α, p‐STAT3‐Tyr, p‐STAT3‐Ser and STAT3 expression levels were assessed by Western blotting. Representative result of 3 independent experiments. Quantification plots are shown on the right. Data are represented as mean ± SEM. *P < .05, **P < .01, one‐way ANOVA followed by Tukey post‐test in comparison with control. #*P* < .05, ##*P* < .01, one‐way ANOVA followed by Tukey post‐test in comparison with CoCl_2_ or 1% O_2_ samples

To confirm the clinical relevance of HIF‐1α and STAT3 interaction, we collected 20 tumour samples from TNBC patients. Strong colocalization of HIF‐1α, p‐STAT3‐Tyr and p‐STAT3‐Ser in the nucleus was observed (Figure 1A,1B). Furthermore, we analysed mRNA data of The Cancer Genome Atlas (TCGA) breast cancer cohorts and found that HIF1A expression was significantly overexpressed in 1215 breast cancer specimens compared to 113 non‐tumour tissues (Figure 1C) and positive correlations were observed between HIF1A and STAT3 (Figure 1D) (r = 0.1998,* P* < .0001).

Next, we established both CoCl_2_ and hypoxia chamber models and evaluated the phosphorylation of STAT3 at both tyrosine and serine sites. Cooccurrence activation of HIF‐1α with p‐STAT3‐Tyr and p‐STAT3‐Ser was found in MDA‐MB‐231 cells (TNBC) (Figure 1E). We next sought to natural compounds which can inhibit breast cancer cells via HIF‐1α and STAT3 pathways. We found sanguinarine (IC_50_ = 5.2 μM) could attenuate HIF‐1α, p‐STAT3‐Tyr and p‐STAT3‐Ser expression in hypoxic MDA‐MB‐231 cells under hypoxic conditions (Figure 1F).

### 3.2 Sanguinarine inhibited colocalization and interaction of HIF‐1α and p‐STAT3

**Figure 2:**
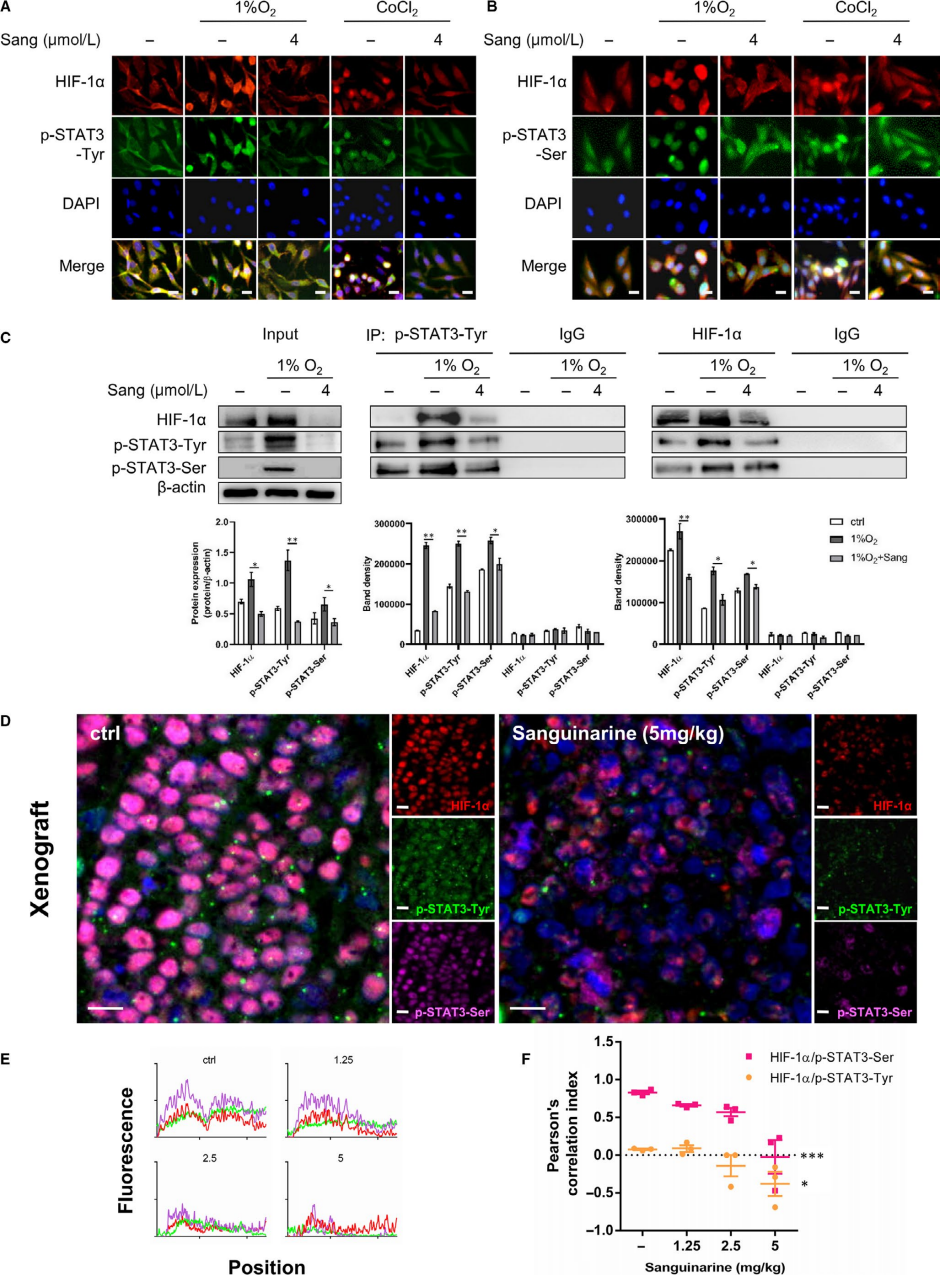
Sanguinarine inhibited colocalization and interaction of HIF‐1α and p‐STAT3. A, MDA‐MB‐231 cells were treated with 4 μM sanguinarine in the absence or presence of 1% O_2_ for 12 hours. B, MDA‐MB‐231 cells were treated with 4 μM sanguinarine in the absence or presence of CoCl_2_ for 12 hours. HIF‐1α (red), p‐STAT3‐Tyr (green), p‐STAT3‐Ser (green), DAPI (blue) staining and merged images indicated the nuclear localization. Scale bar, 10 μm. C, MDA‐MB‐231 cells were treated with different concentrations of sanguinarine in the absence or presence of 1% O_2_ for 24 hours. Cell lysate was immunoprecipitated with anti‐p‐STAT3‐Tyr, anti‐HIF‐1α or IgG, HIF‐1α, p‐STAT3‐Tyr and p‐STAT3‐Ser expression were assessed by Western blotting. 10% input shows results obtained from cell extracts without immunoprecipitation. Quantification plots are shown below. *P < .05, **P < .01, one‐way ANOVA followed by Tukey post‐test in comparison with or 1% O_2_ samples. D, MDA‐MB‐231 xenograft tumour samples were stained for HIF‐1α (red), p‐STAT3‐Tyr (green), p‐STAT3‐Ser (magenta), DAPI (blue). Scale bar, 10 μm. E, Plot profile of immunofluorescence pictures from control and 1.25, 2.5, 5 mg/kg sanguinarine‐treated xenograft samples were analysed by ImageJ. F, Pearson's correlation indexes of HIF‐1α/p‐STAT3‐Ser and HIF‐1α/p‐STAT3‐Tyr were evaluated with immunofluorescence pictures from MDA‐MB‐231 xenograft samples

In MDA‐MB‐231 cells, 1% O_2_ incubation and CoCl_2_ caused colocalization of HIF‐1α with p‐STAT3‐Tyr or p‐STAT3‐Ser. Sanguinarine could effectively disrupt the colocalization and lead to the distribution of HIF‐1α and p‐STAT3 to the cytoplasm (Figure 2A,2B). HIF‐1α/p‐STAT3 interaction in hypoxic MDA‐MB‐231 cells was precipitated by p‐STAT3‐Tyr or HIF‐1α antibodies. Interestingly, HIF‐1α interacted with both p‐STAT3‐Tyr and p‐STAT3‐Ser, which was also affected by sanguinarine (Figure 2C). To assess the effect on colocalization of HIF‐1α, p‐STAT3‐Tyr and p‐STAT3‐Ser by sanguinarine in vivo, the colocalization in MDA‐MB‐231 xenograft was examined by immunofluorescence. Notably, strong colocalization was observed in tumour tissue from the control group, whereas sanguinarine treatment significantly altered the subcellular localization of p‐STAT3‐Tyr and especially p‐STAT3‐Ser (Figure 2D), and potently inhibited the colocalization of HIF‐1α/p‐STAT3‐Tyr and HIF‐1α/p‐STAT3‐Ser (Figure 2E, 2F).

## 4 DISCUSSION

Hypoxia results from increased O_2_ consumption and decreased oxygen availability due to rapid dividing and structurally and functionally abnormal vessel formation within solid tumours. In breast cancer, the median tumour oxygen level is only 1.3%.^10^ The activity of HIFs precisely regulates vital biological processes via transcriptional activation of more than 100 downstream genes.^4^, ^11^ HIFs modulate genes involved in angiogenesis, stem cell maintenance, metabolic reprogramming, epithelial‐mesenchymal transition, invasion, metastasis and resistance in breast cancer progression.^12^ Indeed, high levels of HIF‐1α commonly found in breast cancers especially TNBC are associated with high patient mortality.^13^ Recent studies demonstrated that HIF‐1α knockdown in human breast cancer cells slows primary tumour growth and decreases metastasis in mice bearing breast tumours.^14^ Therefore, targeting HIF‐1α could be a potential option to treat breast cancer. HIF‐1α translocates to nuclei to activate a number of hypoxia‐responsive genes after binding to the promoters or enhancers of target genes. STAT3 is one of the transcription factors specifically required for HIF‐1α target gene induction.^15^

In this study, we demonstrate that HIF‐1α colocalizes with p‐STAT‐Tyr and p‐STAT3‐Ser in TNBC patient tissues. Consistently, in vitro hypoxic models revealed that in TNBC cells HIF‐1α interacts with both p‐STAT‐Tyr and p‐STAT3‐Ser leading to the induction of target proteins. Notably, we found that sanguinarine could effectively disrupt HIF‐1α/STAT3 colocalization. Intriguingly, we also found in TNBC xenograft models that sanguinarine treatment altered STAT3 phosphorylation pattern at tyrosine and serine sites differently. p‐STAT3‐Tyr levels were decreased while p‐STAT3‐Ser nuclear localization was disturbed by sanguinarine. Immunostaining of TNBC patient samples and co‐IP results confirmed that HIF‐1α, p‐STAT3‐Tyr and p‐STAT3‐Ser compose the transcriptional complexes during hypoxia. Importantly, our findings demonstrated that sanguinarine disrupted the formation of the complex. This offers one potential mechanism by which sanguinarine may contribute to breast cancer inhibition. Our results may bring insights to the HIF‐1α/STAT3 interaction in breast cancers and suggest sanguinarine may potentially be recognized as HIF‐1α/STAT3 targeted compound for disturbing the growth of human breast cancers.

## CONFLICT OF INTEREST

The authors declare no conflicts of interest.

## AUTHOR CONTRIBUTION

QS, JW and MF performed the experiments and analysed the data. MG, AU and BW provided technical and material support. BD, YZ and DZ contributed essential reagents or tools and supervised all the experimental procedure. QS and YZ designed the project, wrote and revised the manuscript. All authors read and approved the final manuscript.

## ETHICS APPROVAL AND CONSENT TO PARTICIPATE

All procedures and experiments involving animals and patients were approved by the biomedical ethics committee of Xi'an Jiaotong University Health Science Center and conform to ethical principles. The reference number is 2019‐1032.

## Data Availability

All data generated or analysed during this study are included in this article. The data that support the findings of this study are also available on request from the corresponding author.
