# Supplementary material for: Sanguinarine disrupts the colocalization and interaction of HIF‐1α with tyrosine and serine phosphorylated‐STAT3 in breast cancer
Source: J Cell Mol Med. 2020 Feb 17;24(6):3756–61. doi: 10.1111/jcmm.15056 (PMC7131922; doi:10.1111/jcmm.15056)
Supplement: Supplementary file 1 [file JCMM-24-3756-s001.docx]

**Sanguinarine disrupts the colocalization and interaction of HIF-1α with tyrosine and serine phosphorylated-STAT3 in breast cancer**

Qi Su, Jingjing Wang, Mengying Fan, Mohsin Ahmad Ghauri, Asmat Ullah, Bo Wang, Bingling Dai, Yingzhuan Zhan, Dongdong Zhang, Yanmin Zhang*

School of Pharmacy, Health Science Center, Xi’an Jiaotong University, Xi’an 710061, P.R. China

**Running Title**: Sanguinarine inhibits HIF-1α/STAT3 interaction

**E-mail:**

Qi Su suqi201803@xjtu.edu.cn

Jingjing Wang wjj1714712855@stu.xjtu.edu.cn

Mengying Fan fan950208@stu.xjtu.edu.cn

Mohsin Ahmad Ghauri Ghaurimohsin@stu.xjtu.edu.cn

Asmat Ullah asmatullah@stu.xjtu.edu.cn

Bo Wang logo-wang@163.com

Bingling Dai dbl1412@xjtu.edu.cn

Yingzhuan Zhan zyzlt2009@xjtu.edu.cn

Dongdong Zhang ddzhang@xjtu.edu.cn

Yanmin Zhang zhang2008@mail.xjtu.edu.cn

**Correspondence to:**

Dr. Yanmin Zhang

**Address:** School of Pharmacy, Health Science Center, Xi’an Jiaotong University, No.76, Yanta West Street, #54, Xi’an, Shaanxi Province 710061, P.R. China

**E-mail:** zhang2008@mail.xjtu.edu.cn (YM. Zhang)

**Supplementary Information**

**MATERIALS AND METHODS**

**Chemicals and Reagents**

Sanguinarine (purity ≥ 99%, Lot: HS026156198) was purchased from Baoji Kerui Biochemical Pharmaceutical Co., Ltd (Shaanxi, China). DMEM, RPMI1640, L15, MTT, trypsin, DMSO were purchased from Sigma-Aldrich (St. Louis, MO, USA). Penicillin and streptomycin were obtained from Harbin general pharmaceutical factory (Heilongjiang, China) and North China pharmaceutical (Hebei, China), respectively. Fetal bovine serum (FBS) was purchased from Excell Bio (Shanghai, China). Antibodies against HIF-1α and β-actin were obtained from Protein technology Group (Chicago, Illinois, USA) and antibodies against STAT3, Phospho-STAT3-Tyr and Phospho-STAT3-Ser were purchased from Cell signaling (Boston, Massachusetts, USA). All the antibodies were used in 1:1000 dilution for western blotting. RIPA lysis buffer was purchased from Applygen Technologies (Beijing, China). Protease and phosphatase inhibitor cocktail were purchased from Roche Technology (Basle, Switzerland). Cy3-goat anti-rabbit, CoraLite488-goat anti-rabbit and CoraLite594-goat anti-mouse IgG, BCA protein assay reagent kit and enhanced chemiluminescent (ECL) plus reagent were purchased from Pierce Biotech (Rockford, Illinois, USA).

**Cell lines and cell culture**

Human breast cancer cell line MDA-MB-231 were obtained from Genechem Co.,Ltd. (Shanghai, China). MDA-MB-231 were cultured in L15 medium with 10% (v/v) FBS, supplemented with penicillin and streptomycin. Cells were maintained at 37 °C in a humidified incubator.

**Western blotting**

After treatment, cells were lysed in RIPA buffer containing phosphatase and protease inhibitors for 30 min at 4 °C. Total protein content was measured by BCA assay. The protein samples were loaded to SDS-PAGE and transferred to a PVDF membrane (Millipore, Bedford, MA, USA), which was blocked with 5% BSA and incubated with the indicated primary antibodies at 4°C overnight. Then the membrane was incubated with species-specific secondary antibodies and protein expression were visualized with the enhanced ECL. The images was obtained by Tanon5200 imaging system (Tanon, Shanghai, China).

**Immunofluorescence**

Human TNBC samples were obtained from Shanghai Zhuolibiotech company Co., Ltd. (Shanghai, China). The study has been approved by the biomedical ethics committee of Xi'an Jiaotong University Health Science Center and conforms to ethical principles (2019-1032). Samples were block with 3% BSA. Afterwards, samples were incubated with primary antibodies against HIF-1α (1:1000), p-STAT3-Tyr (1:1000), p-STAT3-Ser (1:200) at 37°C for 4 h.

Cell samples were fixed with 4% paraformaldehyde and then blocked with 10% BSA for 30 min at room temperature. Afterwards, tumor samples and the cells were incubated with primary antibodies against HIF-1α (1:200), p-STAT3-Tyr (1:200), p-STAT3-Ser (1:200) at 37°C for 4 h. Then, samples were incubated with an Cy3-conjugated anti-rabbit, [CoraLite488](http://www.ptgcn.com/products/CoraLite488-%E2%80%93-conjugated-Affinipure-Goat-Anti-Rabbit-IgG-H-L--secondary-antibody.htm)-conjugated anti-rabbit and CoraLite594-conjugated anti-mouse secondary antibody (1:50) at room temperature for 60 min. The nucleus was stained with DAPI. Fluorescent images were taken by inverted fluorescence microscope (DM505, Nikon Co., Ltd., Otawara, Tochigi, Japan). Colocalization profile and Pearson's correlation index were analyzed using ImageJ software.

**Cell viability assay**

Cells (1×10^4^ cell/well) were seeded into 96-well plates and treated with different concentrations of sanguinarine for 48 h and 0.5 mg/mL MTT for additional 4 h. After the removal of medium, 150 μL DMSO was added to each well. The plates were placed on a shaker for 15 min and then analyzed using a microplate reader (Bio-Rad, Hercules, CA, USA) at 490 nm.

**Animals and xenograft models**

All the experiments were in accordance with the guidelines of the Institutional Animal Care and Use Committee. All procedures and experiments involving animals and patients were approved by the biomedical ethics committee of Xi'an Jiaotong University Health Science Center and conforms to ethical principles. The reference number is 2019-1032. 200 μL MDA-MB-231 cell suspension (2 ×10^7^ cells/mL) was subcutaneously implanted into the right axillary of the nude mice (female immune-deficient BABL/c nude mice). Mice were randomly divided into control group and treatment groups (n=5) when the tumor volume reached around 100 mm^3^. Mice in the control group were orally administered with 0.5% sodium carboxymethyl cellulose (CMC-Na) solution or different concentrations (1.25, 2.5, 5 mg/kg) of sanguinarine. Animals in every group received ten times of drug administration for consecutive three days and every other day during which the body weight and tumor volume were recorded. The mice were sacrificed on the last day, tumors were removed and fixed in 4 % paraformaldehyde. Tumor specimens were embedded in paraffin and cut into 5 μm sections for immunofluorecent staining.

**Co-immunoprecipitation**

Cell extracts were prepared from cells following the protocol of immunoprecipitation kit KIP-2 (Proteintech) and an equivalent amount was used for co-immunoprecipitation. Cell lysates were immunoprecipitated with rabbit anti-phospho-STAT3-Tyr, anti-HIF-1α antibody or rabbit anti IgG isotype control and incubated overnight at 4 °C. 10% input was the non-immunoprecipitated extracts.

**Statistical analysis**

One-way analysis of variance (ANOVA) and further Dunnett’s multiple comparison test and t-test were used and *P* value <0.05 was considered statistically significant (Prism 6.0, GraphPad, La Jolla, CA, USA). Data are presented as mean±S.E.M.
